# Incidence and Pathological Patterns of Nephrotic Syndrome among Infants and Children: A Systematic Review

**DOI:** 10.7759/cureus.58331

**Published:** 2024-04-15

**Authors:** Shehab A Alenazi

**Affiliations:** 1 Department of Pediatrics, Faculty of Medicine, Northern Border University, Arar, SAU

**Keywords:** a systematic review, incidence, pediatric nephrology, nephrotic syndrome, children

## Abstract

Nephrotic syndrome (NS) is known to be a prevalent chronic illness in young patients. Periorbital swelling in children with this condition is a recurring symptom, either with or without generalized edema. The current study aimed to examine the incidence and pattern of nephrotic syndrome in infants and children by thoroughly examining the recently available literature. A thorough search of PubMed, SCOPUS, Web of Science, Science Direct, and Google Scholar was conducted, following the Preferred Reporting Items for Systematic Reviews and Meta-Analyses (PRISMA) model, to find pertinent material. The Rayyan software (Qatar Computing Research Institute, Ar-Rayyan, Qatar) was utilized during the whole process. Data from a total of 1418 patients from nine trials were considered in this study. Numerous factors influenced the incidence, mean age, sex dominance, and histological patterns in various sample groups. The current findings conclude that variations in socioeconomic, regional, and genetic factors influence the development and pattern of these diseases. The prevalence of pediatric renal disorders differs throughout countries. Season of occurrence, response to corticosteroid treatment, and histopathologic findings appear to differ amongst the diagnosed cases.

## Introduction and background

The clinical condition known as nephrotic syndrome (NS) is characterized by excessive proteinuria that causes hypoalbuminemia, which in turn causes hyperlipidemia, edema, and other problems. Increased permeability through the damaged basement membrane in the renal glomerulus is the reason, particularly in cases of infectious or thromboembolic disease. In children, it is characterized by protein excretion exceeding 40 mg/m^2^/hour. A urine protein/creatinine ratio of 2-3 mg/mg or higher in the morning in both adults and children is indicative of nephrotic-range proteinuria.

NS is caused by an anomaly in glomerular permeability, which can be due to congenital infections, diabetes, systemic lupus erythematosus, neoplasia, or specific drug use, or it might be the primary outcome of an intrinsic renal illness in the kidneys [1,2]. People of all ages are susceptible to the illness. Facial edema is typically the initial presentation of NS in most children. Congenital NS can be classified into congenital NS which is manifested between birth and three months, infantile NS among infants between three and 13 months, and childhood NS which presents in ages 12 months or older. [1].

NS can affect children of any age. However, in boys in particular, the illness most commonly affects children between the ages of two and seven years [3]. The most typical symptom of NS in children is swelling around the eyes [4]. The swelling is typically worse in the morning and, if it's mild, it could be mistaken for seasonal allergies. Primary NS and secondary NS are the two types of NS that affect children.

In children and adolescents, primary NS can be categorized into different forms of kidney disease [4]. Minimal change disease (MCD), is the commonest cause of NS among young children. Focal segmental glomerulosclerosis (FSGS) is the second most common cause, which is characterized by scarring of the kidney's glomeruli with a higher incidence of occurrence of renal failure. Membranous nephropathy (MN) is a widely reported form caused by an autoimmune condition that results in an accumulation of immunological proteins in the glomerular basement membrane of the kidney.

Children's secondary NS can be caused by systemic disorders, which are illnesses affecting multiple organs or the body as a whole. Some of the conditions that fall under this category are lupus, IgA vasculitis (also called Henoch-Schönlein purpura), infections such as hepatitis B and C, HIV, and malaria, blood diseases like leukemia, lymphoma, and sickle cell disease, as well as certain medications and drugs like nonsteroidal anti-inflammatory drugs (NSAIDs) and some medications used to treat mood disorders, bone loss, or cancer [3].

A medical and family history, physical examination, urine tests, blood tests to assess kidney function, and an examination for underlying diseases are all used in the diagnosis of NS in children. Kidney biopsy, genetic testing, and ultrasonography are possible further tests to determine the etiology of NS. A kidney biopsy is not necessary for many children with NS. Children with complex diseases, those with impaired kidney function, and those who are 12 years of age or older are typically the only ones eligible for kidney biopsy [4].

The cause of NS determines the appropriate course of treatment. As such, the way adult and pediatric populations are managed differs. Children with idiopathic NS are the primary patients for corticosteroids. For children with steroid-dependent NS or those who relapse frequently, alternative immunosuppressive medications are frequently required. In the pediatric population, the anti-B cell antibody rituximab has been shown to be a successful steroid-sparing medication. However, in children who are dependent on both steroids and calcineurin inhibitors, rituximab may not be able to induce drug-free remission. Rituximab might also be useful for children with NS who are resistant to steroids [3].

## Review

Methods

The current systematic review research methodology followed the widely acceptable Preferred Reporting Items for Systematic Reviews and Meta-Analyses (PRISMA) guidelines [5].

Study Design and Duration

The review research was carried out in September of 2023. To locate the pertinent literature, a comprehensive search was conducted across five main databases: PubMed, SCOPUS, Web of Science, Science Direct, and Google Scholar. We limited our search to English publications and took into account the particular needs of every database. To locate the pertinent papers, the following keywords were transformed into PubMed Medical Subject Headings (MeSH) terms: "nephrotic syndrome," "infants," "incidence," "prevalence," and "diagnosis." The necessary keywords were matched by the Boolean operators "OR" and "AND". Among the search results were publications with full text in English, freely downloadable articles, and human trials.

Selection Criteria

The articles included in the current review were only case-control studies and randomized control trials (RCTs) examining the diagnosis and treatment of NS in children and infants (less than 18 years old) carried out between 2004 and 2023. Only free articles available in the English language were selected for further analysis.

Data Extraction

The search strategy's output was checked for duplication using the Rayyan software (Qatar Computing Research Institute, Ar-Rayyan, Qatar) [6]. The combined search results were modified using a set of inclusion/exclusion criteria in order to evaluate the titles and abstracts for relevancy. Then each paper was examined carefully with regard to the fulfillment of the inclusion requirements. Techniques to resolve data conflicts were applied. A data extraction form based on the previously published articles was used to upload the approved study. The extracted data include study titles, authors, year of study, country, gender, participants, purpose of study, and outcomes. Then the risk of bias was assessed using a separate sheet. The risk of bias was assessed using the Risk of Bias in Non-randomized Studies of Interventions (ROBINS-I) to evaluate the quality of the enrolled studies fulfilling the inclusion criteria [7].

Strategy for Data Synthesis

A qualitative overview of the findings was produced by creating summaries of the study components and findings based on data from relevant research. Once the data for the systematic review were acquired, the most efficient way to use the data from the included study articles was chosen.

Results

Search Results

**Figure 1:**
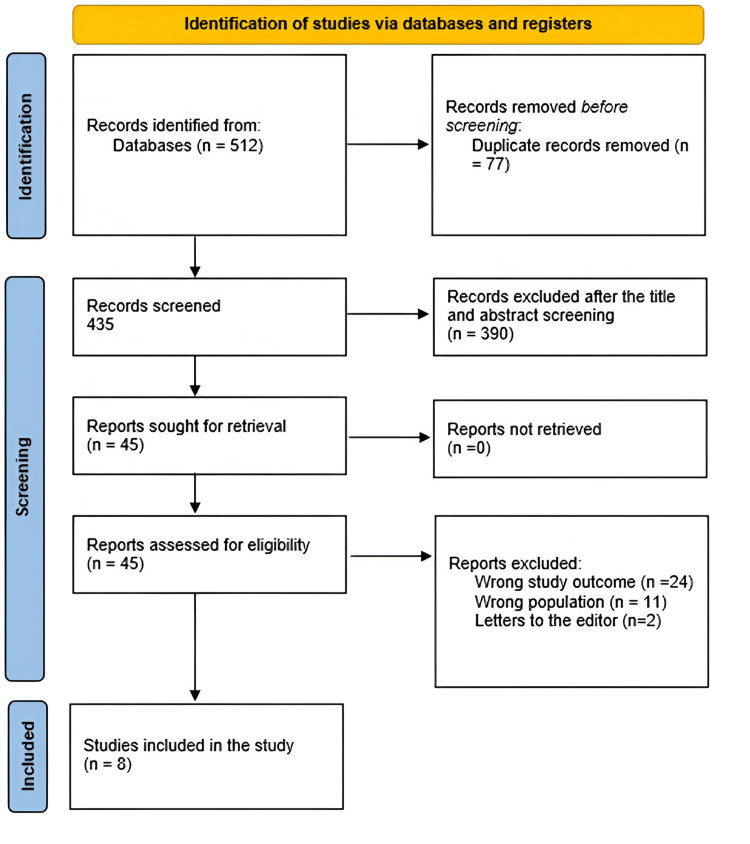
Flowchart summarizes PRISMA steps for the selection process of the studies enrolled for analysis. PRISMA: Preferred Reporting Items for Systematic Reviews and Meta-Analyses

A total of 512 study papers were found from the systematic search. Of these, 77 duplicates were removed. A total of 390 papers were determined to be ineligible for inclusion after the remaining 435 studies were subjected to title and abstract screening. A total of 45 articles were eventually examined for full-text assessment, from which 24 were excluded for having the wrong study outcomes, 11 for having the wrong population type, and two for being letters to the editor. There were finally eight study articles that qualified for this systematic review. A summary of the research selection process is shown in Figure 1.

The sociodemographic characteristics of the included enrolled articles are shown in Table 1 including study design, country, and the number, age, and gender (male) of participants. Total number of children enrolled in the selected studies was 1418. Seven studies fulfilling the inclusion criteria were retrospective while only one study was prospective. Study locations include Germany, Egypt, Iran, and Pakistan with the remaining four studies conducted in four different regions in Saudi Arabia. The mean age of the studied groups varied from 4.3 to 9.83 years. Male participation was higher in five studies with nearly equal percentages between males and females in two studies while one study showed lower participation of males.

**Table 1:** Sociodemographic characteristics of the included studies Our results included eight studies with 1418 patients NM: not mentioned

Study	Study design	Setting	Number of participants	Mean age (years)	Male participants, n (%)
Franke et al., 2018 [8]	retrospective	Germany	346	5.5 ± 3.7	224 (64.7%)
Alharthi, 2016 [9]	retrospective	Taif. Saudi Arabia	87	5.8	45 (51.7%)
Alhassan et al., 2013 [10]	retrospective	Aljouf, Saudi Arabia	25	6.2	17 (68%)
Abdullah, 2012 [11]	retrospective	Western Saudi Arabia	242	NM1	122 (50.4%)
Kari et al., 2012 [12]	retrospective	Jeddah, Saudi Arabia	36	4.3 ± 3.0	11 (30.5%)
Kaddah et al., 2011 [13]	retrospective	Egypt	100	9.84 ± 3.9	74 (74%)
Safaei and Maleknejad, 2009 [14]	prospective	Iran	44	NM	29 (66%)
Mubarak et al., 2009 [15]	retrospective	Pakistan	538	9.79 ± 4.59	347 (64.4%)

Regarding the aim of the selected articles, studies were mainly targeting the prevalence and histopathological pattern of NS among diagnosed children as well as pattern of therapeutic response as shown in Table 2.

**Table 2:** Aims and conclusions of the included studies NS: nephrotic syndrome; SRNS: steroid-resistant nephrotic syndrome; SDNS: steroid-dependent nephrotic syndrome; SSNS: steroid-sensitive nephrotic syndrome; FSGS: focal segmental glomerular sclerosis; MPGN: membranoproliferative glomerulonephritis; MCD: minimal change disease; MesPGN: mesengioproliferative glomerulonephritis; C1qNP: C1q nephropathy

Study	Aim	Main Outcomes	ROBIS-!
Alharthi, 2016 [9]	To describe the various histological features and clinical characteristics of NS in Taif region-affected children, as well as how they responded to treatment.	According to this study, 70% of patients respond to steroid medication, which is almost in line with the findings of several other studies. Thirty percent of individuals with SSNS had steroid dependency, and 17% had relapses often. The three main histological patterns of childhood SRNS in the Taif region were FSGS, MPGN, and MCD nephropathy. The significant consanguinity rate in our patients explains the high frequency of SRNS and SDNS.	High
Alhassan et al., 2013 [10]	To ascertain the frequency and occurrence of the various NS patterns in kids in our area of Saudi Arabia, as well as how well they respond to treatment.	There were no notable differences between the NS patterns and treatment response seen in this study and those conducted in other parts of the world.	Moderate
Abdullah, 2012 [11]	In a sizable academic center in the western part of Saudi Arabia, to evaluate the prevalence and distribution of every histological category of renal disorders in the pediatric age range (birth to 17 years old).	Due to the previously listed factors, the incidence of different nephropathies, particularly glomerulopathies in the pediatric age range, differs both within and between our country and other countries. NS is the most prevalent clinical indication for renal tissue sampling in this study, and glomerulo-nephritis is the most commonly found disease	Moderate
Kari et al., 2012 [12]	King Abdul Aziz University Hospital in Jeddah, Saudi Arabia, to report on the pattern of SRNS histopathology in pediatric patients.	When children presented with SRNS, the most prevalent underlying histopathology was FSGS. In a few other children, the underlying cause was IgM nephropathy and other types of MCD, including MesPGN and C1qNP.	High
Kaddah et al., 2011 [13]	To record the response to various therapy and examine the epidemiological, clinical, and histological characteristics of Egyptian children with idiopathic NS.	Compared to patients in other studies, a higher proportion of steroid-resistant patients was observed in this patient population. Their response to immunosuppressives varied from other research, most likely as a result of variations in the importance of immunosuppressive therapy selection.	Moderate
Safaei and Maleknejad, 2009 [14]	To evaluate the range of nephrotic syndrome in children in Iran.	The timing of the incidence, the seasons at the time of clinical presentation, the histological results of biopsies, and the appropriate response to corticosteroid treatment are all different from those of other studies carried out in Iran and other nations.	Moderate
Mubarak et al., 2009 [15]	To ascertain the pattern of glomerulopathies using renal samples examined by light microscopy, immunofluorescence microscopy, and electron microscopy.	Overall, MCD and its variations are the most common cause of INS in children, with FSGS, the major pathology in SRNS and teenage NS, following closely behind. Our observations are consistent with similar biopsy indications from recent international series. For the first time in Pakistan, the study identifies the actual pattern of glomerulopathies in pediatric INS patients.	Moderate

Discussion

A frequent glomerular condition in children, NS is characterized by severe proteinuria and edema. NS in children increases the risk of chronic kidney disease, complications from the condition, and side effects from therapy. The majority of NS cases are of the primary, idiopathic type, which is frequently brought on by a banal infection in infancy or early childhood. There are alternative therapeutic avenues but cortisone therapy administered over several weeks is currently the standard course of treatment [16].

According to current reports, the incidence of NS varies among different population groups and is between two and seven per 100,000 children [17]. Numerous earlier published data found the incidence rate, mean age, and sex preponderance. Males make up 74% of those with childhood NS, according to Kaddah et al. [13]. In their study, the mean age at which the condition started was 4.43 ± 2.7 years, and 81% of patients had a disease start that was less than six years old. In additional research, the patients with onset ages ≤ 6 years varied in proportion from 46% to 79%, and the mean age of onset ranged from 4.6 to 5.4 years [12].

According to a prior study, children with NS have a male-to-female ratio of about 1:1 [9]. There is no difference in the gender rate between children with SSNS or SRNS, according to similar findings from a recent study comparing the gender rate between boys and girls. In contrast, a different study on NS in Saudi Arabian children revealed a 2.1:1 male-to-female ratio among young patients. According to other studies, the male-to-female ratio of 1.6 to 2.7:1 indicates that the majority of young children affected by this disease are male. Even so, by the time puberty arrives, there is no longer any gender disparity in the rate between males and females in adolescents and adults [9]. 

Previously reported data indicate that the epidemiology of NS in infants and children varies greatly based on the underlying reasons. According to Mubarak et al., MCD and its variations account for 43.8% of occurrences of INS in children, making them the primary cause of the condition [15]. With MCD present in 33.5% of cases, it was the most common among them. IgM and C3 diffuse mesangial positivity and mild to moderate mesangial hypercellularity were observed in 10.3% of patients. According to an American study by Srivastava et al., 52.7% of biopsies revealed MCD [18]. Regionally, MCD was found in 34.2% and 32% of children according to two Indian studies [19,20].

Patients with NS typically experience substantial protein loss in their urine, which causes hypoproteinemia and edema. There is also a correlation with hyperlipidemia, hypercholesterolemia, and elevated lipiduria [20,21]. In contrast to previous investigations, the participants in the study by Safaei and Maleknejad presented with distinct signs and symptoms [14]. Also, the frequency of hypertension and microscopic hematuria in Indian children was 26.8% and 41.8%, respectively [22,23].

FSGS is the most prevalent histological subtype (41%), among the children who underwent biopsies [14].Turkish children's biopsies revealed pathologic features consistent with mesangial proliferate glomerulonephritis in 49% of cases [23,24]. In contrast, Indian children's most common histological subtype was FSGS (38%)[20].

The prevalence of FSGS has been observed to range from 15% in a study by Ei-Reshaid et al. [25] in Kuwait to 59% in the study by Gulati et al. [26] in India. The primary etiology of SRNS in Kuwaiti children was reported to be MCD (65%) in the report [25]; however, in Kari et al.'s study, it contributed just 8% to SRNS [12]. These findings are consistent with earlier investigations that found FSGS to be the primary underlying histology in children with SRNS [27-33].

Abdullah conducted a similar study which found that the most common clinical indication in the study group (n=242) was NS (n=117, 48.3%) [11]. This was followed by renal mass for histological diagnosis (n=17, 7.0%), systemic lupus erythematosus for staging (n=30, 12.4%), and nephritic syndrome (n=27, 11.1%). Acute renal failure (n=2, 0.8%), congenital nephritic syndrome (n=2, 0.8%), hydro-nephrosis (n=4, 1.6%), transplant kidney diseases (1.6%), hematuria (5.4%), end-stage renal disease (5.4%), obstructive uretro-pelvic junction diseases and other congenital kidney diseases (2.5%), and proteinuria of the non-nephrotic range (2.5%) were among the others. This result is consistent with previously published data [28,29].

With a proportion of 75.6%, glomerulopathies, particularly the primary ones, account for the majority of juvenile renal disorders that are recorded. Following this are vascular renal diseases/glomerulosclerosis (4.1%) and renal neoplasms (5.8%). This result is consistent with data from Deshpande et al. from India, who reported that primary glomerular disease accounted for 61.5% of all nephropathies and was the most frequent entity [32]. However, studies from other nations, like Tunisia [33] and Iran [34], highlight some distinctions, listing congenital urologic malformations, including reflux nephropathies, obstructive uropathy, and dysplastic kidneys, as the most prevalent, as well as hereditary nephropathies. Remarkably, a more recent study carried out in Saudi Arabia revealed that FSGS accounts for almost 62% of all SRNS biopsies performed on children, making it the most common histology. This finding is consistent with other research conducted in Saudi Arabia and other regions, which suggests that the primary underlying histology in children with SRNS was FSGS [9].

A systematic treatment program consisting of glucocorticoids (steroids) successfully treats an initial episode in about 90% of cases, which are steroid-sensitive [34]. Nevertheless, relapses occur in around 80% of these patients [35,36]. Of these, half regularly experience relapses or are classified as steroid-dependent [37]. Steroids can treat any relapse [38], although children may be more susceptible to the negative consequences of a large cumulative dose of steroids [39].

NS course and steroid response differ depending on ethnicity and geographic location. Many regions of sub-Saharan Africa have historically been reported to have significant rates of steroid resistance. More recent research indicates that the percentage of steroid-sensitive NS cases is rising, with some centers even nearing European estimates [40,41]. Twenty-nine (66%) of the 44 children in the Safaei and Maleknejad study were steroid sensitive, nine (20.5%) were steroid resistant, and six (13.5%) were steroid dependent [14]. Two individuals developed DMP and seven patients had FSGS among those with NS resistant to steroids. Studies conducted elsewhere have reported 76-87% varying corticosteroid sensitivity [3].

In contrast to most other studies, where steroid resistance ranged from 9.4% to 22%, certain investigations found that 34% of patients developed steroid resistance following their initial prednisone medication [13].

## Conclusions

Worldwide, a wide range of renal disorders, such as glomerulopathies, tubulinterstitial, vascular, congenital, and neoplastic lesions, affect children under the age of 17. NS is one of the most common and serious kidney conditions among children. Due to variations in socioeconomic, regional, and genetic factors that influence the development and pattern of these diseases, the prevalence of pediatric renal disorders differs throughout countries. Season of occurrence, response to corticosteroid treatment, and histopathologic findings appear to differ amongst the diagnosed cases. Also, it has been shown that the pattern of NS lesions may vary with time in the same region. This highlights the importance of continuous research on NS to track changes that may affect the therapeutic plan as well as the prognosis of the diagnosed cases
